# Phylogenetic and Trait-Based Assembly of Arbuscular Mycorrhizal Fungal Communities

**DOI:** 10.1371/journal.pone.0036695

**Published:** 2012-05-14

**Authors:** Hafiz Maherali, John N. Klironomos

**Affiliations:** 1 Department of Integrative Biology, University of Guelph, Guelph, Ontario, Canada; 2 Department of Biology, I.K. Barber School of Arts and Sciences, University of British Columbia – Okanagan, Kelowna, British Columbia, Canada; Cinvestav, Mexico

## Abstract

Both competition and environmental filtering are expected to influence the community structure of microbes, but there are few tests of the relative importance of these processes because trait data on these organisms is often difficult to obtain. Using phylogenetic and functional trait information, we tested whether arbuscular mycorrhizal (AM) fungal community composition in an old field was influenced by competitive exclusion and/or environmental filtering. Communities at the site were dominated by species from the most speciose family of AM fungi, the Glomeraceae, though species from two other lineages, the Acaulosporaceae and Gigasporaceae were also found. Despite the dominance of species from a single family, AM fungal species most frequently co-existed when they were distantly related and when they differed in the ability to colonize root space on host plants. The ability of AM fungal species to colonize soil did not influence co-existence. These results suggest that competition between closely related and functionally similar species for space on plant roots influences community assembly. Nevertheless, in a substantial minority of cases communities were phylogenetically clustered, indicating that closely related species could also co-occur, as would be expected if i) the environment restricted community membership to single functional type or ii) competition among functionally similar species was weak. Our results therefore also suggest that competition for niche space between closely related fungi is not the sole influence of mycorrhizal community structure in field situations, but may be of greater relative importance than other ecological mechanisms.

## Introduction

Functional traits have long been hypothesized to influence community assembly because organism function determines the ability to tolerate climatic conditions, acquire resources and interact with other individuals [1]–[4]. When functional traits are shared by closely related species (i.e., conserved), phylogenies can be used to determine whether organism function has played a role in the assembly of a given community [1], [4]–[7]. For example, if environmental filtering influences community assembly, then co-occurring species should share characteristics that enable survival in a particular habitat. As a result, communities would be phylogenetically clustered, or more closely related than expected by chance. If competition influences community assembly then co-occurring species should not share functional characteristics, resulting in communities that are phylogenetically even, or more distantly related than expected by chance. Because traits may or may not be conserved, phylogenies may not necessarily be effective proxies for assessing similarities in the functioning of closely related species. Therefore, both trait and phylogenetic perspectives are necessary to test hypotheses about the relative effects of environmental filtering and competition on the assembly of communities [6], [7].

Though phylogenetic or trait information has been used to examine community assembly [6], these perspectives have been combined in only a small number of cases [5], [8]–[10]. Moreover, studies that combine phylogenetic and trait information have been confined to communities of macro-organisms such as plant and animals. Nevertheless, communities of micro-organisms can also be structured by processes such as environmental filtering and intense competition among closely related species [11]–[13]. Microbial species strongly influence ecosystem processes as well as the performance of plants and animals, but hypotheses about how functional trait evolution influences community assembly are more difficult to test than with macro-organisms because of a lack of information on the traits that define microbial niches [12].

In this study, we employ phylogenetic and trait-based approaches to test hypotheses about mechanisms of community assembly in the field for an ecologically important phylum of microbes, the arbuscular mycorrhizal (AM) fungi (Glomeromycota). A majority of species are in three distinct taxonomic families (Glomeraceae, Acaulosporaceae, and Gigasporaceae) within two orders (Glomerales and Diversisporales) [14]. AM fungi are an ancient lineage of obligate biotrophs which must form associations with plants in order to obtain energy for growth and reproduction [15]. AM fungal communities are known to respond to variation in climate, soil resources and plant host identity [16]–[18], as well as influence plant function [19], [20] and the coexistence of plant species [21]. Despite their ecological importance, little is known about the mechanisms that regulate community assembly in AM fungi [22].

Functional traits associated with spatial niches are similar among closely related species in the Glomeromycota (i.e., they are conserved) [23], [24]. For example, members of the Gigasporaceae extensively colonize soil but exhibit limited and slow colonization of roots. Conversely, species in the Glomeraceae rapidly and extensively colonize roots but produce limited hyphal biomass in soil. The Acaulosporaceae form a third distinct group that tend to be poor colonizers of both soil and roots. Because of this trait conservatism, phylogenies can be used to test hypotheses about the mechanisms of community assembly in AM fungi. By experimentally manipulating the phylogenetic relatedness of AM fungal communities under uniform host and soil conditions, we have previously shown that realized species richness was highest when the starting species were more distantly related to each other and did not share similar functional traits [20]. However, fungal communities in the field are likely to be influenced by dispersal limitations, priority effects, host variation, soil heterogeneity and stochasticity [16], [25], all of which may supersede trait and phylogenetic effects. Thus, field studies are necessary to determine the relative importance of various ecological mechanisms responsible for community assembly.

To determine whether phylogenetic and trait dispersion influence AM community composition under field conditions, we examined the species composition of AM fungal communities in an old field. If closely related and functionally similar species compete, it would be expected that communities would be phylogenetically even and consist of species with dissimilar trait values. If soil conditions or plant hosts act as habitat filters, it would be expected that communities would be phylogenetically clustered and consist of species with similar trait values. We tested these predictions by sampling soil at regular intervals within a 50 m×50 m grid. We characterized AM fungal community composition at each sampling point based on the morphological identification of spores. We calculated whether species composition at each sampling point was phylogenetically even or clustered and whether trait dispersion was greater or lower than expected by chance.

## Materials and Methods

### Site Description and Plot Layout

We established survey plots at the Long-Term Mycorrhiza Research Site (LTMRS), an old field meadow dominated by perennial herbaceous plants, and which is located on relatively even ground in the Nature Reserve of the University of Guelph Arboretum, Guelph, ON (43°32′30″ N, 80°13′00″ W). Soils at the site are generally nutrient poor, and particularly low in phosphorus (2.1 mg P kg^−1^ dry soil) [26]. Though the site has been used for agriculture in the past, cultivation was abandoned in 1967. In 2000, we placed a single 50 m×50 m gridded plot in the centre of the site. Previous analyses suggest that because of limited spore dispersal [15], AM fungal community composition is spatially structured at scales <50 cm [27], [28]. Therefore, we established sampling points on the grid at 1 m intervals (51×51 points = 2601 community samples).

### Sampling Species Richness

We used trap cultures to determine the species richness of AM fungal communities. Though trap cultures can exclude species that have poor rates of colonization or specific host requirements [22], [29], previous research suggests that they nevertheless capture relatively high numbers of species [18]. In addition, trap cultures allowed us to include only those species that were sporulating, avoiding bias associated with the inclusion of ecologically inactive resting spores in whole soil samples [22]. Our previous results indicate that estimates of species richness obtained from 18 s rRNA-based terminal restriction fragment length polymorphism (t-RFLP) analysis were positively correlated with known species richness in trap cultures [20]. The morphological species identifications using trap cultures were also necessary to match taxa with AM fungal species cultured from the same field site that had been used to obtain trait information [20].

Each grid point was marked as the center for a plot. To characterize the species richness of the community at each grid point, we sampled species located within a 30 cm radius of the center point. We placed four stakes 30 cm from the center in each cardinal direction. In June, we collected 4 soil subsamples using a soil corer (3 cm in diameter, 15 cm deep), which were then pooled and mixed well. Our previous research suggests that closely related AM fungal species can compete to colonize plant roots [20], raising the possibility that the trap culture technique could filter species in a way that produces phylogenetically even communities. To reduce the likelihood that competition for root space would restrict the taxa recovered from soil samples, we established three separate trap cultures for each sampling point. We note that other methods of limiting competition in trap cultures are available, such as using low amounts of inoculum to initiate cultures. However, we opted to divide soils into multiple trap cultures to increase the likelihood of root colonization and sporulation. AM fungal species lists for each sampled community consisted of species pooled across the 3 trap cultures.

To establish trap cultures, we divided the soil sample into three parts and placed it in a Cone-tainer (SC10, Stuewe & Sons, Tangent, OR, USA) which had the bottom 2/3 filled with a mix of 50% inert calcined clay (Turface, Profile Products LLC, Buffalo Grove, IL, USA) and 50% silica sand. The top 1/3 of the container was filled with field soil. The resulting 7803 Cone-tainers were randomly placed on benches in the greenhouse. To provide abundant root area for fungal colonization, five seeds of leek (*Allium porrum*), a species that is frequently used as a general host for AM fungal species [30], were added to each Cone-tainer, and thinned to three plants per pot after germination. Plants were watered daily and no fertilizer was added.

To minimize the possibility that life history differences in spore germination and growth rate would bias taxon recovery from soil samples, trap cultures were harvested after 12 weeks, providing enough time for species from different AM fungal families to colonize roots [23], . At harvest, plant shoots and the top 1/3 of the pot containing the field soil were removed. The bottom 2/3 of the pot, which contained the growth medium, leek roots and freshly produced spores, was used to extract and identify AM fungal species. These materials were mixed in a blender, suspended in water, and then passed through a series of sieves whose mesh ranged from 1 mm, 0.5 mm, 0.3 mm, and 0.047 mm. The fraction remaining on the smallest sieve size was placed in a beaker and decanted twice to remove heavy particles that settled to the bottom. The floating fraction was placed on a wet nitrocellulose filter and sealed in a petri dish. We mounted up to 100 AM fungal spores on slides with 1∶1 (v/v) of polyvinyl-alcohol-acetic-acid-glycerol and Melzer’s Reagent [30] and identified them using morphological and developmental characters as described on the International Culture Collection of Vesicular Arbuscular Mycorrhizal Fungi (INVAM) web site (http://invam.caf.wvu.edu/fungi/taxonomy/speciesID.htm). Because spore abundance depends strongly on life history [15], it was not an appropriate metric for quantifying species abundance. As a result, species were scored as either present or absent. Grid points where no species were found were eliminated from the analysis of community assembly.

To describe the spatial distribution of each species based on the presence or absence at each sampling point, we calculated the Morisita index of dispersion (*I*_δ_) [31]. Because *I*_δ_ requires information on abundance, we carried out this analysis using species presence data aggregated over 4 adjacent sampling points (i.e., derived from 2×2 m plots). As a result, the maximum abundance each species could have in the analysis was 4 (e.g., a species was found at each of the 4 sampling points included in each aggregated plot). *I*_δ_ values <1 indicate repulsion among individuals, manifested as an even distribution; *I*_δ_ ∼1 suggests a random distribution; and *I*_δ_ >1 indicate attraction among individuals, manifested as clumping. *I*_δ_ could not be calculated for the rarest taxon, *Scutellospora pellucida*, which was found at only 6 points on the sampling grid.

### AM Fungal Trait Data

To determine whether AM fungal traits influenced community composition, we obtained information on the extent of root and soil colonization from previous studies of the same fungal taxa collected at the same site [20]. AM fungi were cultured by inoculating seedlings of *Plantago lanceolata* with single fungal spores of each species. These cultures were grown for one year in 20 cm diameter pots containing sterilized field soil. After cultures were established, 50 g of AM fungal inocula were added to pots containing sterilized field soil along with a germinated seedling. After 1 year of growth, root colonization (percentage of root length infected [32]) and soil hyphal length (m hyphae g^−1^ soil [33]) were measured. Though fungal traits and performance can vary with plant host [15], our previous studies suggest root colonization and soil hyphal length were similar when assessed using four old field species as hosts [24]. Therefore, we assumed that these fungal traits were representative of the performance of each species, rather than being an outcome of specific interactions between the host plant and fungal species.

### Phylogenetic Tree Construction and Analyses of Trait Conservatism

To determine whether shared evolutionary history could explain patterns of species coexistence, we developed a phylogenetic tree using previously published molecular phylogenies [24], [34], [35]. Because these phylogenies were created with different gene sequences, we manually pruned and combined these trees to produce a topology that included only the taxa found in our old field sample plot. Because of this method of tree construction, branch lengths were not available. Therefore, we set all branch lengths to 1, a conservative assumption that minimizes type I error rate in comparative analyses [36].

To verify that fungal traits were conserved [24] using the species found at our field site, we calculated contribution indices (CIs) for each node in the phylogeny and a tree-wide phylogenetic signal using the ‘aotf’ function in PHYLOCOM 4.2 [37]. Contribution indices vary between 0 and 1 and estimate the degree to which individual nodal divergences along the phylogeny contribute to extant trait variation [38]. A trait was considered conserved if significant variation is explained more by relatively ancient than recent divergences in the phylogeny. Phylogenetic signal is derived from the tree-wide variance of standardized independent contrasts [39]. If closely related lineages have similar traits, then the magnitude of the independent contrasts should be low, resulting in low tree-wide variance. To determine if CIs and tree-wide phylogenetic signal were statistically significant (*P*≤0.05), they were compared to a distribution of 1000 values calculated by randomly swapping trait values across the tips of the phylogeny in PHYLOCOM [37]. This method also generates randomized trait values at internal nodes because character reconstruction is based on the randomized tip values.

### Phylogenetic and Trait-based Analyses of Community Composition

To test whether phylogenetic relationships and functional traits influenced AM fungal species assemblages, we compared phylogenetic relatedness and observed patterns of trait variation for each of the sampled communities to randomly generated communities derived from a ‘constrained’ null model that assumes that the probability of a species contributing to an assemblage is determined by its overall frequency across the entire sampling grid [37], [40]. The null communities were created by randomly swapping species occurrences among all sampling grid points while maintaining the species richness of the observed community at each sampling grid point [41]. Although other null models were available for comparison to sampled communities [40], we used the constrained null model for several reasons. First, this null model was developed for species presence/absence data, which made it suitable for our study design. Second, simulations suggest that in groups such as AM fungi, where both traits [24] and species frequency [42] are conserved, this null model is less prone to Type 1 error [43]. Third, the spatial distribution for a majority AM fungi at our site was clumped (see results). The constrained null model preserves some degree of this spatial autocorrelation, which reduces Type 1 error rate in tests of trait and phylogenetic-based community composition [44]. All analyses were done using PHYLOCOM 4.2 [37].

To quantify the phylogenetic relatedness of co-occuring species, we calculated the mean nearest phylogenetic taxon distance (MNTD) using the ‘comstruct’ function in PHYLOCOM. MNTD is defined as the average distance to the closest relative of each species in the sample [37]. Communities that are phylogenetically even have MNTDs higher than expected by chance, whereas communities that are phylogenetically clustered have MNTDs lower than expected by chance. We chose MNTD over other relatedness metrics [6] because simulations indicate that in situations where functional traits are conserved, this metric is most suitable for detecting phylogenetic evenness and clustering [45] while minimizing Type I error rates [43].

To quantify trait variation within each community, we calculated the variance (VAR) of root colonization and soil hyphal length for both observed and null communities using the ‘comtrait’ function in PHYLOCOM. If competition structures communities, then species with dissimilar traits are more likely to co-occur, and trait variance in observed communities should be higher than that generated in null communities. Conversely, if habitat filtering influences species assemblages, then species with similar traits are more likely to co-occur, and trait variance in observed communities should be lower than that generated in null communities.

To determine whether MNTD and trait variance in an observed community differed statistically from MNTD and trait variance in a randomly assembled community, we compared observed values to a distribution of 9999 communities generated from the null model. To determine whether the observed community MNTD or trait variance was significantly different from the null community using a two tailed test (α = 0.05) we calculated whether it was in the top or bottom 2.5% of the null distribution (i.e., 250/10000).

To examine whether the overall pattern in MNTD and trait variance across the sampling grid was consistently different from null expectations, we calculated a standardized effect size (SES) for each metric for each sampling point based on the difference between the observed metric and the mean metric of the null communities. For MNTD, we calculated the nearest taxon index (NTI) as: NTI = −1×[(MNTD_OBS_−MNTD_RANDOM_)]/sd(MNTD_RANDOM_). A negative NTI indicates that a community is phylogenetically even, whereas a positive NTI indicates that a community is phylogenetically clustered. For trait variation we calculated SES_VAR_ as: SES_VAR_ = [(VAR_OBS_−VAR_RANDOM_)/sd(VAR_RANDOM_)]. A positive SES_VAR_ indicates that traits are dispersed in a community whereas a negative SES_VAR_ indicates that traits are clustered within a community. We used one sample Wilcoxon signed ranks tests to determine if the site level distributions of NTI and SES_VAR_ for each trait were significantly different from a null expectation of zero.

## Results

### Fungal Richness and Frequency

**Figure 1:**
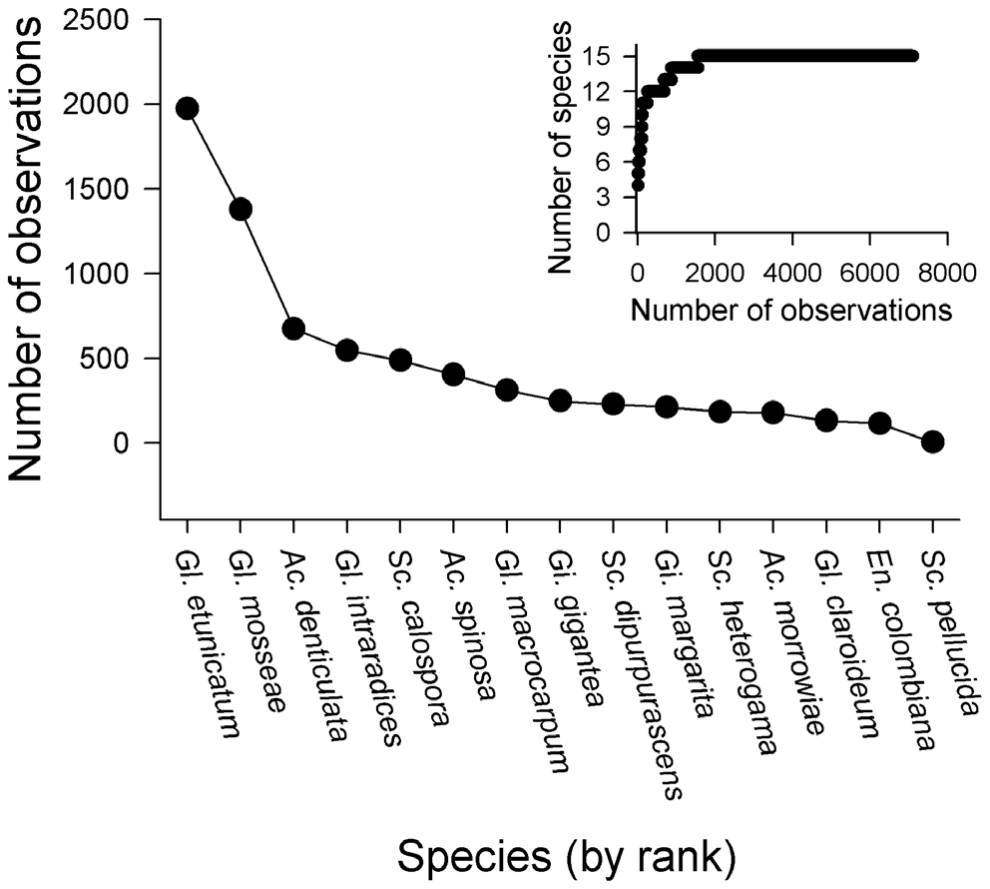
The frequency of AM fungal species across the 50 m×50 m sampled grid and a species rarefaction curve (inset).

**Figure 2:**
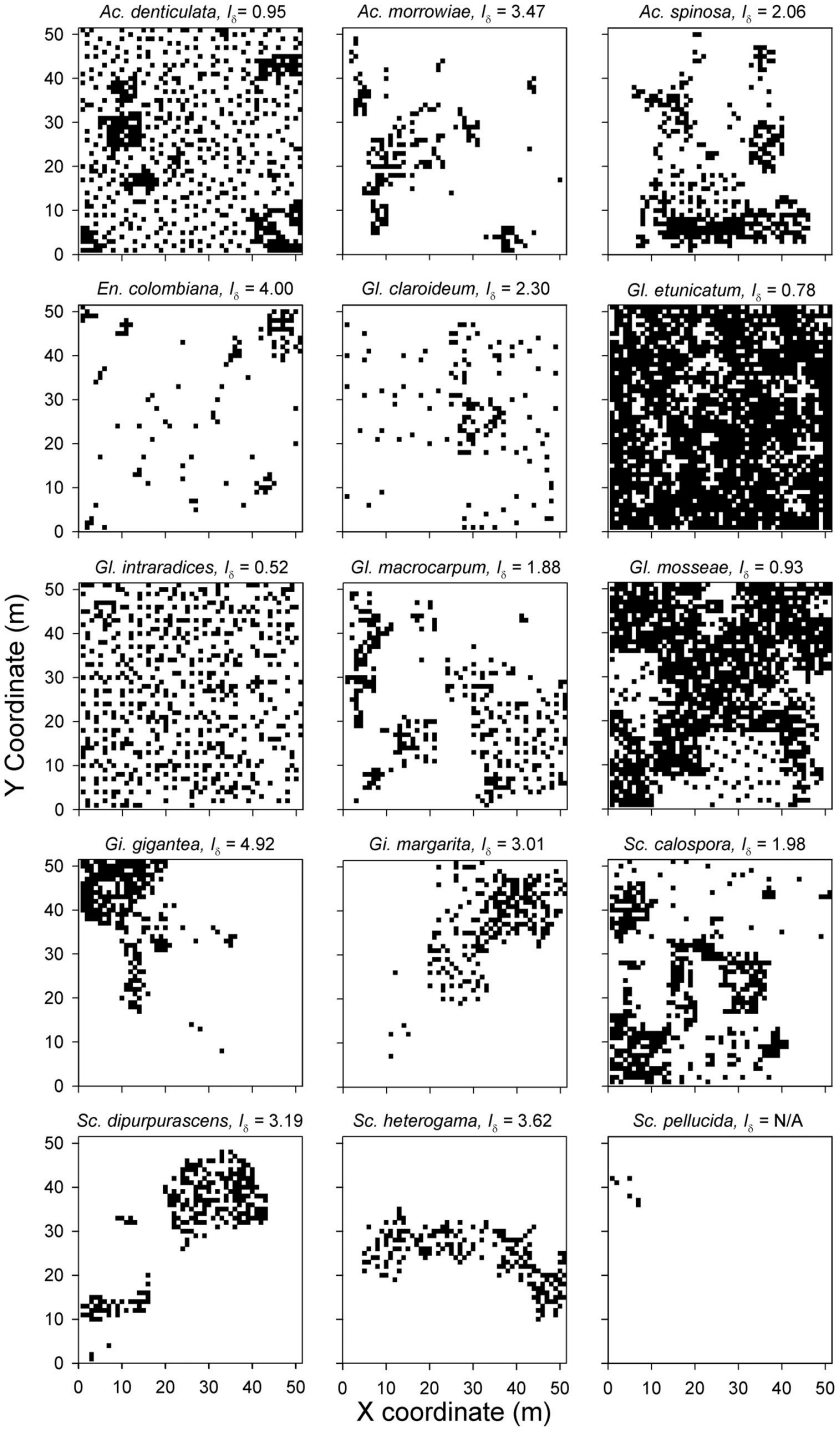
The distribution of each species across the 50 m×50 m sampling grid. Species were scored as present or absent in each of the 2601 sampled communities.

There were sporulating AM fungal species in 2532 of the 2601 sampling grid points and species richness in these communities ranged from 1 to 8 taxa. There were 2151 communities for which mean nearest phylogenetic taxon distance (MNTD) could be calculated (i.e., where richness was ≥2). A total of 15 species spanning 3 families were identified (Figure 1, 2), 57% were Glomeraceae, 24% were Acaulosporaceae and 19% were Gigasporaceae. Our sampling protocol was sufficient to reach saturation for number of species existing at the site (Figure 1, inset). Ten of 14 species for which Morisita’s index (*I*_δ_) could be calculated had values >1, indicating a clumped distribution pattern (Figure 2). The four most abundant species (Figure 1), however, had values that were ∼1 or <1, indicating either a random or even distribution pattern, respectively.

### Fungal Trait Conservatism

**Figure 3:**
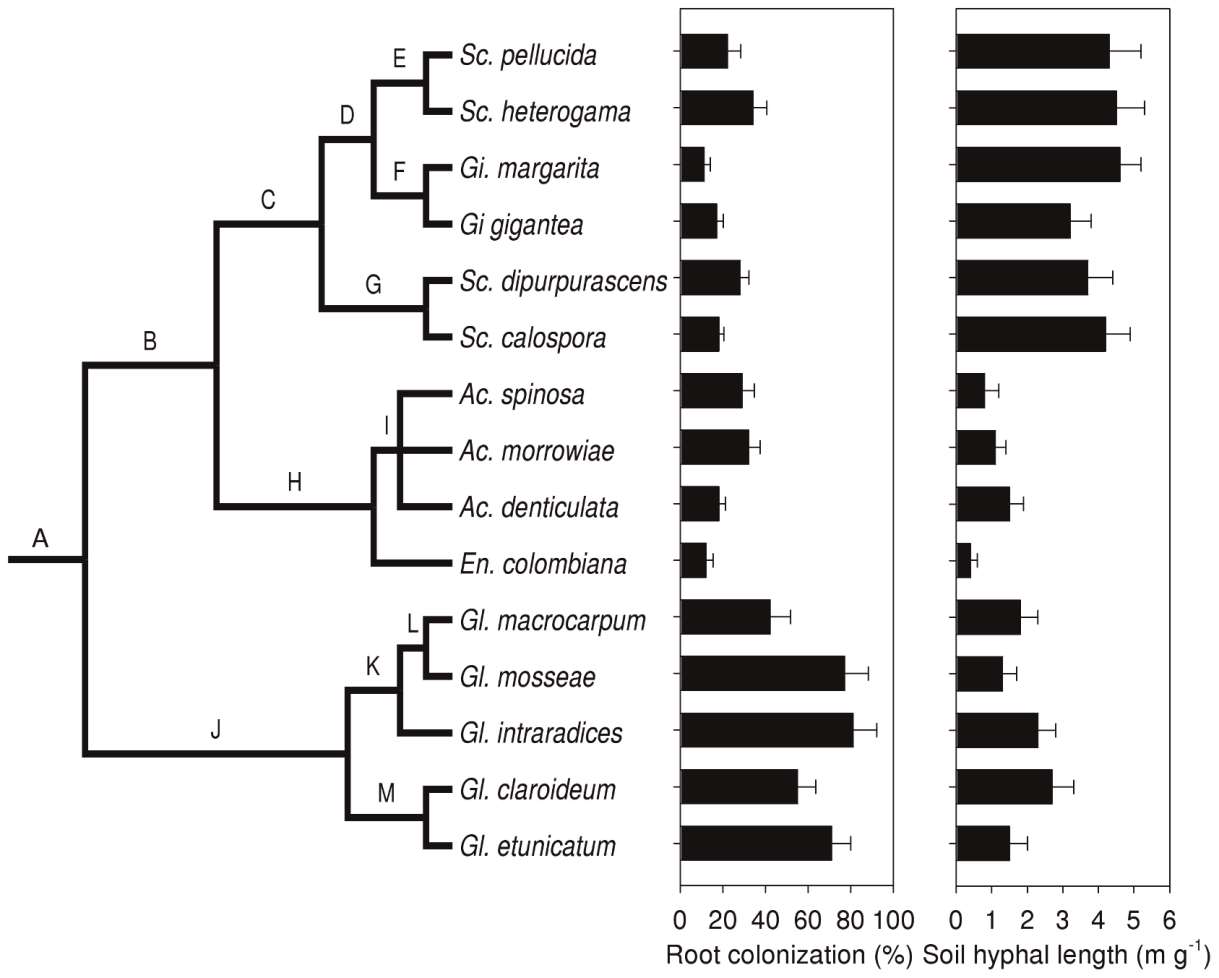
A phylogeny of AM fungi found in the 50 m×50 m sampling grid, along with trait values for Root Colonization and Hyphal Length mapped to each taxon. Both traits were phylogenetically conserved.

AM fungal functional traits were conserved. We detected a significant tree wide phylogenetic signal for the extent of root colonization (contrast variance = 0.497, *P* = 0.002) and hyphal colonization of soil (contrast variance = 163.2, *P* = 0.004). The bulk of extant trait variation was accounted for by deep divergences in the phylogeny (Figure 3). For root colonization, the divergence between the Glomerales and Diversisporales had the largest contribution index (CI) accounting for 83% of extant trait variation (Node A, *P* = 0.001). For hyphal length colonization of soil, the divergence between Gigasporaceae and Acaulosporaceae within the Diversisporales had the largest CI, accounting for 84% of extant trait variation (Node B, *P* = 0.001).

### Phylogenetic and Trait-based Community Assembly

**Figure 4:**
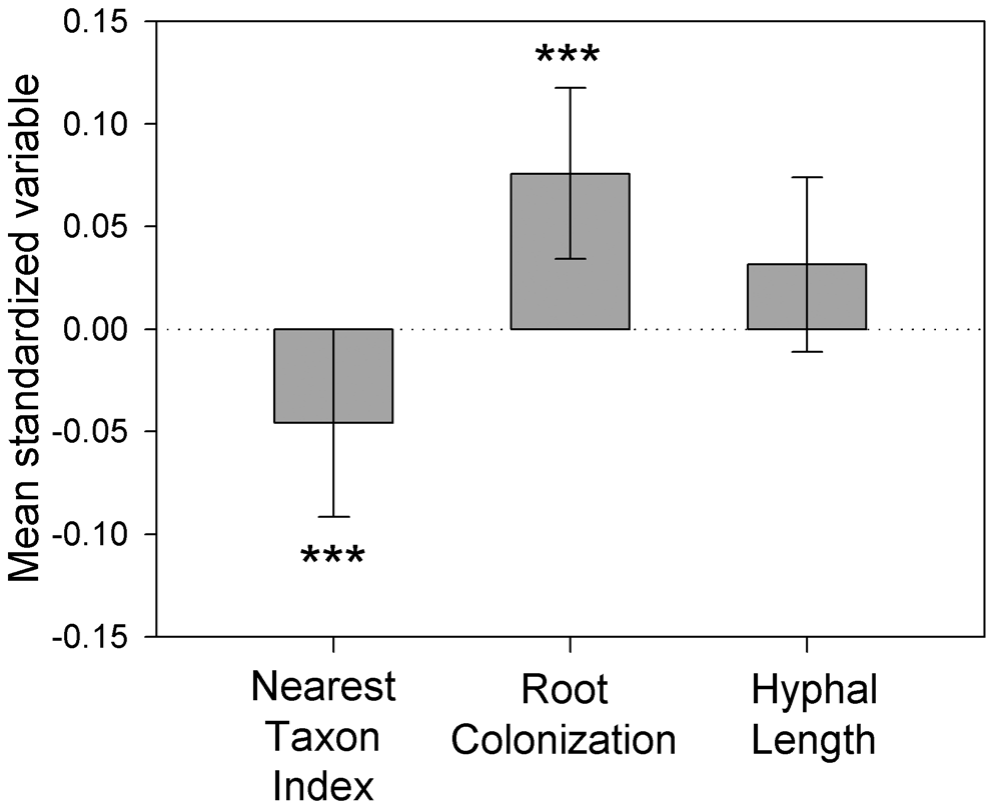
Mean (±95 CI) Nearest Taxon Index (NTI, A), standardized trait variance (SES_VAR_) for Root Colonization (B) and SES_VAR_ for Hypal Length in soil (C) in the 50 m×50 m sampled grid. Mean NTI was significantly lower than 0, indicating that communities were more phylogenetically even than expected by chance. Mean Root Colonization variance was significantly higher than 0, indicating that community trait variance was higher than expected by chance. Mean Hyphal Length in soil did not differ from 0. ***P<0.0001.

AM fungal communities were most frequently phylogenetically even; 36 sampling points had MNTDs significantly higher than expected by chance, whereas 17 sampling points had MNTDs that were significantly lower than expected by chance. The distribution of standardized effect sizes for MNTD, expressed as the Nearest Taxon Index (NTI) was significantly <0 (Figure 4, Test statistic = −4.15, *P*<0.0001) and 54.8% of sampling points had NTIs <0.

The extent of root colonization in AM fungal communities was more often dispersed than clustered; 91 sampling points had trait variances significantly higher than expected by chance, whereas 89 sampling points had trait variances significantly lower than expected by chance. The distribution of SES_VAR_ for root colonization was significantly >0 (Figure 4, Test statistic = 3.56, *P*<0.0001) and 53.1% of sampling points were >0.

The extent of soil hyphal colonization in AM fungal communities was most frequently clustered; 102 sampling points had trait variances significantly lower than expected by chance, whereas 76 sampling points had trait variances higher than expected by chance. Though 58.6% of sampling points had values <0, the distribution of SES_VAR_ for soil hyphal colonization did not differ significantly from 0 (Figure 4, Test statistic = −0.97, *P* = 0.335).

## Discussion

We found evidence for phylogenetic-based community assembly in the AM fungi of an old field. A majority of AM fungal assemblages at our study site were phylogenetically even. This result is consistent with a previous experimental study [20] where we found that AM fungi were more likely to co-exist on roots of a single plant species under uniform soils when they were drawn from different families. Other recent surveys have also shown that AM fungal community composition is non-random [46]. In the current study, fungal communities also had higher than expected variation in the intensity of root colonization. Thus, species that intensively colonize roots were more likely to co-exist with those that had relatively low root colonization. That a majority of communities in the field were made of up of distantly related and functionally dissimilar species suggests that competition for root space influences AM fungal community assembly at small spatial scales, even when other factors such as host identity, soil conditions and dispersal limitations vary in nature [4], [5].

Like previous studies of AM fungal community structure [27], [47], we found that the fungal species assemblage at our study site was dominated by a small number of abundant taxa and that most species had a clumped distribution. In particular, two Glomeraceae species were more frequently observed across the sampling grid than species from other families (Figure 1, 2). This family specific pattern of abundance is consistent with previous local and global surveys using both spore and sequence based sampling techniques, which show that dominant species tend to be members of the Glomeraceae, particularly Group A [18], [42], [48], [49]. Over dominance in AM fungal communities has led to the hypothesis that stochastic processes associated with the opportunistic colonization of roots is a primary mechanism responsible for AM fungal community structure [47]. However, our findings indicate that even when over dominance occurs (Figure 1), competition for root space among functionally similar taxa can still be a determinant of community composition.

Our results differ from a recent global meta-analysis of AM fungal community structure [50] that found that community composition at a site is more frequently phylogenetically clustered than even. This apparent conflict may have arisen from a difference in the phylogenetic scale of the species pool used between studies [51]. To test for the ecological significance of competition at each sampling point in the grid, it was necessary for us to construct null communities assuming that only those species found within the study site were able to colonize any given sampling point, in proportion to their abundance [43], [45]. By contrast, the global meta-analysis tested whether species composition within a site was clustered relative to the global diversity of AM fungi. The difference between the local versus global species pool used for tests of community assembly mechanisms suggests that our findings complement rather than conflict with those done at a global scale. For example, interactions such as competition could determine which species co-exist at small spatial scales within a site, but species composition for the whole site could be restricted by a larger scale ecological filter associated with niche requirements or climate [51].

Though the congruence between phylogenetic evenness and high variation in root colonization intensity within sampled communities suggests that this trait determines co-existence among AM fungi (Figure 4), two other hypotheses could explain the tendency for sampling points to be phylogenetically even. First, other functions that influence fungal fitness such as root colonization rate, spore production rate, frequency of hyphal network formation, uptake of P and N, and the metabolism of sugars [29] could influence co-existence if they are conserved. Testing whether these additional traits influence fungal species co-existence, however, is limited by a lack of information on them in multiple lineages [29]. Second, co-existence in AM fungi could be regulated by negative interactions with consumers, pathogens and parasites [52], [53]. If closely related species share susceptibility to natural enemies [54], then negative density dependence will prevent these species from co-occurring [55]. Testing this prediction is also limited by a lack of information on the extent that AM fungi are regulated by consumers [52], and whether susceptibility to natural enemies is conserved.

Even though hyphal colonization of soil was conserved, this trait was not likely to influence community assembly. Variance in hyphal colonization of soil was lower than for root colonization, and did not differ from zero (Figure 4), suggesting that the dispersion of this trait was random among co-existing species. One explanation for the lack of an association between hyphal colonization of soil and community composition is that AM fungi in the field form mycelial networks through the fusion of hyphae with conspecifics [22] that are likely much larger than in isolated pots [56]. Thus it is possible that hyphal colonization of soil measured on species growing in previously sterilized soil in a pot was not a meaningful indicator of how this trait is expressed in the field. Alternatively, it is also possible that because the volume of soil that can be explored by hyphae is large, there is little competition among AM fungi for this aspect of the niche.

A substantial minority of sampling points contained assemblages that were phylogenetically clustered. This result suggests that environmental filtering also influenced the assembly of AM fungal communities at our study site. One potential cause of filtering is the soil environment, which could affect composition in two ways. First, high nutrient soil patches could eliminate AM fungal species that specialize on nutrient uptake because these functions would not be required by plant hosts [57], [58]. If nutrient uptake capacity is conserved and is higher in specific lineages [20], [24] and these species become extinct in nutrient rich patches, then communities could be phylogenetically clustered. Second, some fungal lineages may have specialized to occupy specific soil texture classes. For example, other field surveys indicate that Glomeracae can dominate on clay soils, whereas Gigasporaceae can dominate on sandy soils [25], [57]. Thus, spatial variation in soil texture could have excluded specific lineages, resulting in sampling points that were phylogenetically clustered. An additional cause of environmental filtering is host identity, which has previously been shown to influence the presence or absence of AM fungal taxa [17], [19], [58]–[60]. Though we lacked information on the identity of roots that AM fungal species associated with, plant species could influence community composition of mycorrhizal fungi in different ways. For example, if the benefits fungi provide a plant species are conserved [24], it is possible that the active culturing or sanctioning of certain lineages by plant hosts in order to maximize that benefit [61], [62] results in communities that contain only one lineage and are therefore phylogenetically clustered. By contrast, if the benefits fungi provide plants are not conserved [63], [64], then culturing or sanctioning by plants could also result in increased phylogenetic evenness of fungal communities.

The occurrence of phylogenetically clustered assemblages could also be caused by the co-existence of closely related species that are weak competitors for niche space [4], [65]. Specifically, species in the Diversisporales (Acaulosporaceae and Gigasporaceae) are closely related and share a low ability to colonize roots. If the poor ability of these species to colonize roots allows them to co-exist, then a substantial number of phylogenetically clustered assemblages should have a mean root colonization value lower than the site median of 52.5%. However, we found that only 13.5% of sampling points had both phylogenetically clustered assemblages and low mean root colonization, a proportion that was significantly lower than expected by chance (*Χ* ^2^ = 11.89, *P* = 0.0006, df = 1). This result suggests that the co-occurrence of weak competitors for root space was relatively rare. However, we also found that a higher than expected proportion of sampling points (31.8%) had assemblages that were both phylogenetically clustered and had mean root colonization values higher than the site median. This finding suggests that closely related species in the Glomeraceae with high root colonization can co-occur frequently (Figure 3). The co-occurrence of these abundant (Figure 1) and closely related species suggests that phylogenetic clustering occurred because of dispersal by dominant species into locations where niche space on roots was not adequately filled [47], [49].

A potential limitation of our study is that sampling AM fungal communities using trap cultures likely resulted in the absence of fungal species that cannot be cultured. Often, molecular methods of AM fungal identification obtain more apparent taxa (operational taxonomic units, or OTUs) than spore based methods [22], and it is therefore likely that total AM fungal species richness was underestimated in our study. Nevertheless, species richness was not outside of the range obtained in molecular-based surveys of old fields and grasslands [16], [47]. In addition, we were able to obtain species from the major families of the Glomeromycota (Fig. 2), suggesting that the trap culture method did not discriminate strongly against specific lineages. Thus, even though species richness was likely underestimated, relative differences in phylogenetic community structure at each sampling point were unlikely to be biased by the trap culture method.

In conclusion, we provide evidence for phylogenetic and trait-based community assembly in AM fungi occurring in a realistic ecological setting. A majority of assemblages at the old field site we sampled were composed of species that were distantly related and differed in the extent to which they were able to colonize roots. Our results were therefore consistent with experimental evidence that competition can prevent functionally similar and closely related taxa from co-existing at small spatial scales [20]. Nevertheless, competition for habitat space on roots was not a universal determinant of the composition of AM fungal communities in the old field, and our results also suggest that habitat filtering can influence community composition at small spatial scales. Moreover, competition may also be weak in certain situations, resulting in communities shaped by the dispersal of abundant species [47], [49]. More generally, our findings suggest that, as in macro-organisms, combining phylogenetic and trait-based approaches can provide insights into the mechanisms of microbial community assembly [12].
